# Protective effect of compound Danshen (*Salvia miltiorrhiza*) dripping pills alone and in combination with carbamazepine on kainic acid-induced temporal lobe epilepsy and cognitive impairment in rats

**DOI:** 10.1080/13880209.2018.1432665

**Published:** 2018-03-21

**Authors:** Chen Jia, Shanshan Han, Liming Wei, Xiangji Dang, Qianqian Niu, Mengyu Chen, Boqun Cao, Yuting Liu, Haisheng Jiao

**Affiliations:** a Department of Pharmacy, Lanzhou University Second Hospital, Lanzhou, China;; b Department of Pharmacy, Huaihe Hospital of Henan University, Kaifeng, China;; c College of Pharmacy, Lanzhou University, Lanzhou, China

**Keywords:** Intractable epilepsy, antiepileptic drugs, traditional Chinese medicine, hippocampal CA3 area, glial cell line-derived neurotrophic factor, anti-apoptosis

## Abstract

**Context:** Temporal lobe epilepsy (TLE) is resistant to antiepileptic drugs (AEDs) and is associated with cognitive impairment. The modern Chinese medicine, compound Danshen dripping pills (CDDP), is clinically effective in treating epilepsy and improving cognitive impairment.

**Objective:** This study evaluated the protective effects of CDDP alone and in combination with carbamazepine (CBZ) on kainic acid-induced TLE and cognitive impairment in rats.

**Materials and methods:** Sprague–Dawley rats were randomly divided into five groups: control (sham operated), model, CDDP, CBZ and combined. A TLE model was then created via bilateral intrahippocampal injection of 0.35 μg kainic acid (KA). Rats received CDDP (85 mg/kg), CBZ (100 mg/kg) or combined (85 mg/kg CDDP +100 mg/kg CBZ) via intragastric administration for 90 d, respectively. Seizure intensity, apoptosis and glial cell line-derived neurotrophic factor (GDNF) were measured. Furthermore, the improvement in cognitive impairment and hippocampal neuronal damage was evaluated.

**Results:** CDDP combined with CBZ significantly decreased seizure severity and frequency (*p* < 0.05) and ameliorated cognitive impairment (*p* < 0.05). The model group showed a significant reduction of neurons and Bcl-2/Bax expression in the hippocampus CA3 area (*p* < 0.01), the combined groups significantly reversed these change (*p* < 0.01). GDNF expression in the combined groups showed a clear increase over the model group (*p* < 0.05).

**Conclusion:** These findings support the use of CDDP as an adjuvant drug for the treatment of TLE and cognitive deficit. Its mechanism might be related to an anti-apoptosis effect and up-regulation of GDNF.

## Introduction

Epilepsy is a terrible chronic neurologic disease of brain dysfunction that is characterized by an enduring state of spontaneous recurrent seizures (SRS); it affects approximately 1–2% of the world’s population (Bell and Sander 2001). Although most types of epilepsy can be effectively controlled by the current antiepileptic drugs (AEDs) such as carbamazepine (CBZ), phenytoin and lamotrigine, 20–30% of patients still suffer from poorly controlled epilepsy and develop intractable epilepsy (IE) (Kwan and Brodie 2002; Riazi et al. 2010; Engel et al. 2012). In these cases, AEDs are unable to influence the long-term harmful effects of status epilepticus or prevent epileptogenesis (Rogawski and Loscher 2004). In recent years, temporal lobe epilepsy (TLE) has become one of the most common forms of IE; patients with TLE are often resistant to medication therapy and are treated in most cases with surgery (Jardim et al. 2012). Because the neuropathy occurs in the memory-relevant brain region, cognitive impairment is a common and severe comorbidity in patients with TLE (Graham and Gaffan 2005; Holmes 2006; Lin et al. 2012). The pathogenesis and drug therapy of TLE has become hotspots in epilepsy research.

Traditional Chinese medicine (TCM) is primarily used as a complementary and alternative medical approach in the treatment of epilepsy. A combination of TCM and Western medicine may increase the curative effect and decrease the recurrence rate and adverse reactions to Western medicine. These combination therapies may offer a new way to treat TLE. Compound Danshen dripping pills (CDDP), consisting of *Salvia miltiorrhiza* Bunge (Labiatae) (known as “danshen” in Chinese), *Panax notoginseng* (Burk.) F.H. Chen (Araliaceae) (known as “sanqi” in Chinese) and borneol are a modern Chinese medicine preparation based on TCM theory and modern preparation technologies. With its multiple targets, components and effects, CDDP is now used extensively in China to prevent and treat diseases including angina pectoris, hyperlipidemia, and coronary heart disease (Chu et al. 2011; Yao et al. 2015). The compound preparation is the first traditional Chinese medicine to complete Phase III clinical trials by the US Food and Drug Administration (FDA). In recent years, some studies have demonstrated several beneficial effects of CDDP. The main component, *Salvia miltiorrhiza*, was found to exert an anticonvulsant effect in a rat model of penicillin-induced epilepsy (Bahadir et al. 2015). Another main component, *Panax notoginseng*, has been shown to have neuroprotective effects against cerebral ischemia/reperfusion injury (Zeng et al. 2014). Clinical studies of patients with epilepsy have shown that combined therapy using CDDP and AEDs is an effective treatment for posttraumatic epilepsy (Jiang et al. 2013). Moreover, compound Danshen tablets, which contain the same ingredients as CDDP, greatly improved cognition impairment in rats with Alzheimer’s disease (Qin et al. 2012; Teng et al. 2014). To date, however, no relevant studies have been published on the protective effects of CDDP in a TLE rat model or the mechanism behind the effects.

Hippocampal neuronal loss is usually a feature of human TLE (Kim et al. 1990). The results of previous studies suggest that spontaneous seizures are initiated in the hippocampal CA3 area (Li et al. 2008), which is characterized by prominent and acute cell loss (Li et al. 2012). Some recent studies have found that apoptosis may lead to seizure-induced hippocampal neuronal loss (Liou et al. 2003; Engel et al. 2010). Various proteins regulate apoptosis, among which proteins of the Bcl-2 gene family, such as Bcl-2 (anti-apoptotic) and Bax (pro-apoptotic), are crucial (Henshall and Simon 2005; Youle and Strasser 2008). In addition, brain-derived neurotrophic factors, such as glial cell line-derived neurotrophic factor (GDNF), play a crucial role in pathologic conditions such as brain damage and seizures (Jankowsky and Patterson 2001). Up-regulation of GDNF has been proposed as a potential antiepileptic mechanism. Hence, this study was designed to assess the effects of CDDP combined with CBZ, one of the most effective and frequently used conventional AEDs, on epileptogenesis and cognitive impairment, and the possible protective mechanism of anti-apoptosis and up-regulation of the GDNF expression level in a model of KA-induced TLE.

## Materials and methods

### Animals

Clean male Sprague–Dawley (SD) rats weighing 240 ± 20 g were purchased from the Laboratory Animal Service Centre of Lanzhou University (animal license SCXY (G) 2013-0002). They were housed at a controlled temperature (22 ± 3 °C) and relative humidity (40%) with a 12 h light/dark cycle. All animal experimental procedures were performed in accordance with the animal care guidelines of the National Institutes for Health and approved by the Animal Ethics Committee of Lanzhou University Second Hospital.

### Experimental design

Seventy male SD rats were randomly divided into five groups (*n* = 14): control (sham operated), model, CDDP, CBZ and combined. Then rats in model, CDDP, CBZ and combined groups were selected to create a TLE rat model by intrahippocampal injection of KA. After the rats were weighed, they were anesthetized with 10% chloral hydrate by intraperitoneal injection (350 mg/kg) and mounted on a stereotaxic instrument (68000 Brain Stereotaxic Instrument, RWD Life Science Co., Ltd, Shenzhen, China). The scalps of the rats were incised to expose the skull surface, and holes about 1.0 mm in diameter were drilled with an incisor bar on each lateral ventricle according to the following coordinates: AP = 0.9 mm anterior to the bregma, ML = ± 2.0 mm from the midline and DV = 5.6 mm below the dura. Then, 0.35 μL of KA (15 mg/kg; Sigma-Aldrich, St. Louis, MO; 1 μg in 1 μL saline solution) was injected into each hole with a micro-syringe. The needle was left in place for at least 5 min. Bone wax was then used to seal the holes and suture the scalp. Rats in the control group were selected to create a sham-operated model by intrahippocampal injection of an equal volume of physiologic saline solution using the same method.

According to the Meeh–Rubner dose equation, clinical doses of CDDP (13.5 mg/kg/d) and CBZ (maximum daily dose 20 mg/kg) for adults are equivalent to CDDP (85.05 mg/kg/d) and CBZ (126 mg/kg/d) for rats. Based on the relevant literature and clinical practice, we chose to give daily doses of CDDP at 85 mg/kg and CBZ at 100 mg/kg by oral administration. The CDDP (27 mg/pill; Tianjin Tasly Co., Ltd., Beijing, China, #151204) and CBZ (0.1 g/pill; Shanghai Zhongxi Pharm. Co., Ltd., Beijing, China, #4080232LA) were ground and dissolved in normal saline solution before use. An HPLC fingerprint of CDDP was shown in Supplemental data.

**Figure 1.**
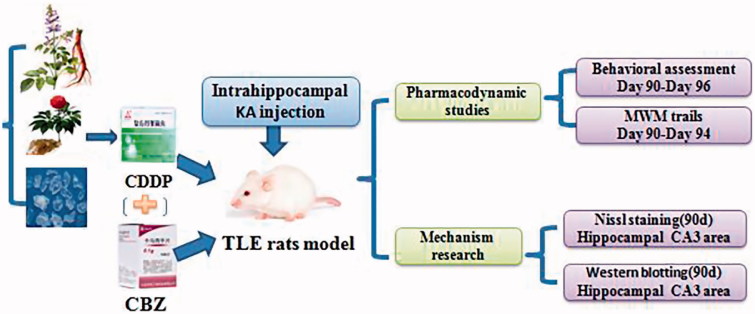
An overview of the experimental design.

After intrahippocampal injection of KA, the rats in each group underwent continuous gavage with the corresponding drug once per day for 90 d. The rats in the control and model groups received physiologic saline solution at 2.5 mL/kg body weight. The rats in the CDDP group underwent gavage with CDDP at 2.5 mL/kg body weight (34 mg in 1 mL of saline solution). The rats in the CBZ group received CBZ at 2.5 mL/kg body weight (40 mg in 1 mL of saline solution). The rats in the CDDP–CBZ combined group underwent gavage with CDDP at 2.5 mL/kg body weight and CBZ at 2.5 mL/kg body weight. The experimental process is shown in Figure 1.

### Behavioral assessment

The rats’ postoperative behaviour was observed over a 9 h period (from 09:00 to 18:00). The Racine stages (Racine 1972) used to assess epileptic seizures in the rats were as follows: 0 = no reaction, normal; 1 = mouth and facial clonus; 2 = head nodding and/or severe facial clonus; 3 = myoclonic jerks in the forelimbs; 4 = rearing with forelimb clonus; 5 = rearing and generalized clonic convulsions associated with rolling and falling. The stages and frequency of SRS (stage 3 and above) in each group were then recorded by an observer.

### Morris water maze (MWM) test

The MWM test (Morris 1984) was used to assess spatial learning and memory behaviour in the epileptic rats (Vorhees and Williams 2006). The test was conducted in a circular pool 100 cm in diameter. The pool was filled with water (21 °C ± 1 °C) to a depth of 26 cm and virtually divided into four quadrants by four equidistant insertion points: NE, SE, SW and NW. A colourless escape platform 10 cm in diameter was submerged about 1 cm below the water surface in the centre of the SW quadrant. The rats were allowed to escape the water using various fixed visual cues present around the pool.

Each test had two parts: a place navigation test and a spatial probe trial. The place navigation test was performed four times a day for four consecutive days of training. The test started by placing a rat at the border of a randomly chosen quadrant other than the SW quadrant and facing the wall of the pool. The test finished when the rat escaped onto the hidden platform and the escape latency was then recorded. Any rat that failed to find the platform within 120 s was guided to the platform and kept there for 15 s; its escape latency was recorded as 120 s. Probe trials without a platform were performed on day 5. The trials allowed the rats to swim freely from the NE quadrant. The platform-crossing frequency and time spent in the NE quadrant were recorded.

### Nissl staining

The rats underwent transcardial perfusion with physiologic saline solution followed by 4% paraformaldehyde for fixation after deep anaesthesia, and their brains were quickly removed and post-fixed over 24 h at 4 °C with 4% paraformaldehyde and transferred into 10%, 20% and finally 30% sucrose solution (Kim et al. 2011). Serial consecutive coronal sections, prepared using a microtome, were mounted on gelatin-coated slides and air-dried overnight. The slides were rehydrated in distilled water and stained in a 1% Toluidine blue solution (Sigma-Aldrich, St. Louis, MO) for 6 min. After rinsing with distilled water, the slides were gradually dehydrated with a series of alcohols, cleared in xylene and cover-slipped. Each stained section was observed with a light microscope (BX-50; Olympus, Tokyo, Japan) to assess the degree of nerve cell loss within the hippocampal CA3 region.

### Western blotting

The brains were quickly removed after the rats were deeply anaesthetized, and the hippocampi were freshly isolated. The total protein was then quickly extracted from the hippocampi using a radioimmunoprecipitation assay buffer containing the protease inhibitor phenylmethylsulponyl fluoride (both from Biyuntian Technologic Inc., Beijing, China). A bicinchoninic acid kit (Biyuntian Technologic Inc., Beijing, China) was used to determine the concentration of the protein. Equal amounts of protein were separated by SDS-polyacrylamide gels, and the protein was transferred onto polyvinylidene fluoride membranes (Millipore, Billerica, MA) at 100 mA for 75 min at 4 °C. Blocking was done with 5% skim milk in Tris-buffered saline solution containing 0.05% Tween 20 (TBST) for 1.5 h at room temperature. The membranes were then incubated overnight at 4 °C with mouse anti-β-actin (1:1000; Zhongshan Biotech., Beijing, China, #TA-09), anti-GDNF (1:100; Abcam, Cambridge, UK, #ab176564), anti-Bcl-2 (1:1000; Abcam, Cambridge, UK, #ab7973) or anti-Bax (1:1000; Abcam, Cambridge, UK, #ab32503) antibodies. The membranes were then incubated with a goat anti-rabbit antibody conjugated to a horseradish peroxidase secondary antibody (1:1000; Zhongshan Biotech., Beijing, China, #ZB2301) for 1.5 h at room temperature. After washing with TBST (3 × 7 min), bands were detected with an enhanced chemiluminescence detection system (Bio-Rad, Hercules, CA). Densitometric analysis was performed using Bio-Rad’s Quantity One software. To determine the relative band density ratio, all values were normalized against β-actin.

### Statistical analysis

SPSS 17.0 software (IBM Corporation, Armonk, NY) was used for statistical analysis. One-way repeated-measures analysis of variance was used for statistical comparisons; Tukey’s test was used for comparisons of two groups. The measurement data were expressed as the mean ± SEM (standard error of the mean). In MWM tests, one-way repeated-measures analysis of variance followed by Tukey’s test was used to evaluate differences in the escape latencies among the groups. A *p* value of less than 0.05 was considered to indicate statistical significance.

## Results

### Seizures induced by intrahippocampal injection of KA

Seizures induced by intrahippocampal KA injection were divided into three stages according to behavioural characteristics: acute phase, incubation period and chronic phase. The acute phase occurred within 24 h of intrahippocampal injection of KA. From 30 min to 2 h after the injections, all rats that had received KA developed status epilepticus, which was characterized by continuous clonic convulsions associated with intermittent rolling and falling, a type of seizure that can cause death in severe cases. After that, the frequency and degree of epilepsy seizures gradually declined and the duration of each epileptic seizure reduced. Finally, the seizures gradually stopped and the rats returned to normal activity. Incubation periods occurred at 2–14 d after surgery. Chronic phases occurred after the incubation period. In this stage, the rats developed SRS and displayed a different Racine stage.

### Effects of CDDP and its combination with CBZ on the degree of seizure

**Figure 2.**
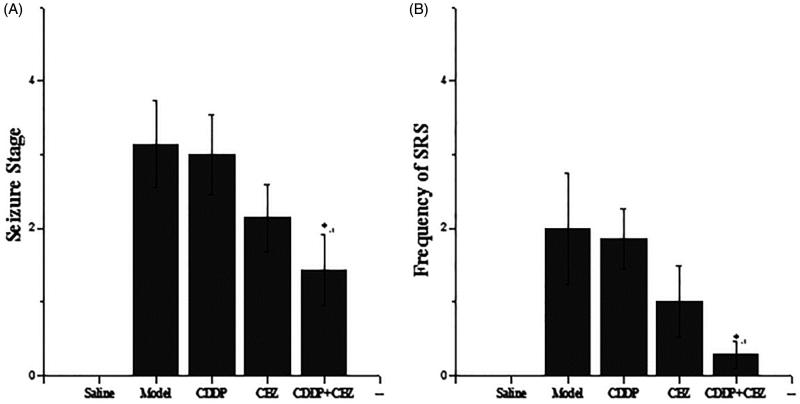
Effects of CDDP and its combination with CBZ on the degree of seizure. (A) Seizure severity score. (B) Frequency of SRS. SRS in each group were recorded three times a day for 1 week, only the seizures of stage 3 or greater according to Racine stages were recorded. The results are presented as mean ± SEM. *p < 0.05 versus the model group (*n* = 14 per group).

To assess the effect of CDDP and its combination with CBZ on the degree of seizure, rats in each group underwent behavioural assessments for 1 week at 90 d after surgery (Figure 2). The saline control rats did not show any spontaneous seizures during the experimental period. Each rat in other groups showed various degrees of seizure ranging from stages 1 to 5 (Figure 2(A)). SRS seizures above stage 3 were observed 0–6 times in the model group rats; 0–6 times in the CDDP group rats; 0–3 times in the CBZ group rats; and 0–1 times in the combined group rats (Figure 2(B)). The combined group showed a significant decrease in seizure stage and the frequency of SRS compared with the model group (*p* < 0.05). Although the degree and the frequency of epileptic seizures in the CBZ and CDDP groups were slightly lower than those in the model group, the results were not statistically significant. Thus, the results show that CDDP in combination with CBZ can effectively control epilepsy.

### Effect of CDDP and its combination with CBZ on cognitive impairment

**Figure 3.**
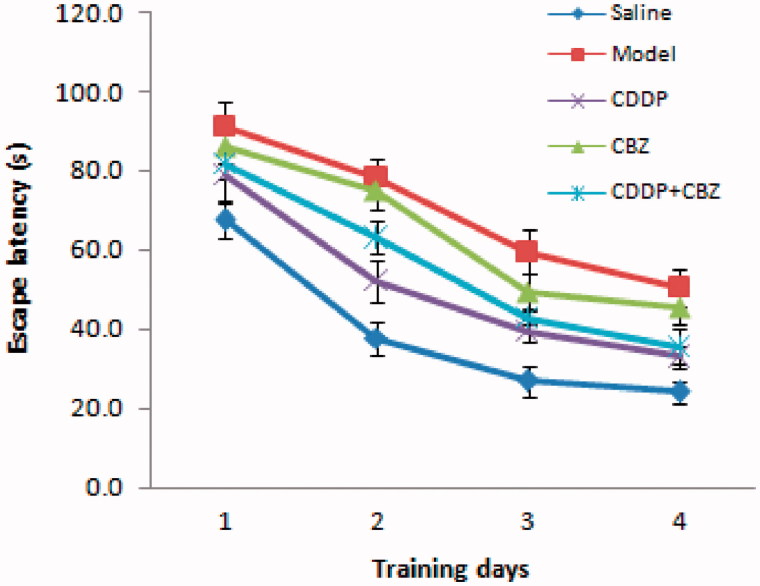
Effect of CDDP and its combination with CBZ on cognitive impairment in the MWM place navigation test. Average escape latencies to find the hidden platform for each trial day are presented as means ± SEM (*n* = 6 per group).

**Figure 4.**
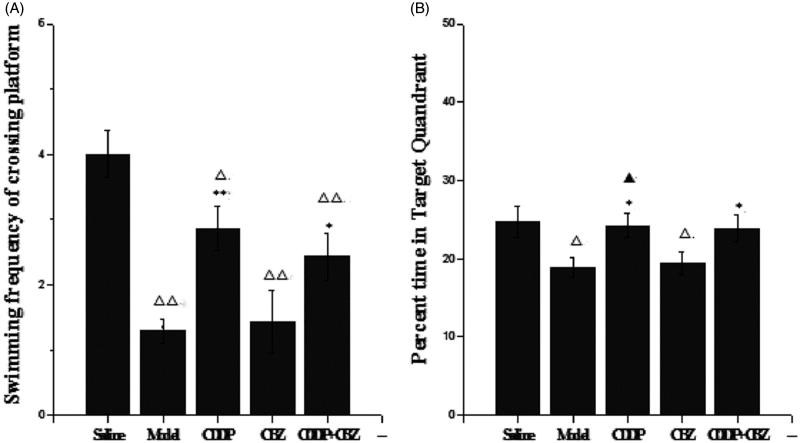
Effect of CDDP and its combination with CBZ on cognitive impairment in the MWM probe trial. (A) Frequency of platform crossing. (B) Time spent in the target quadrant (%). Results are presented as means ± SEM. △*p <* 0.05, △△*p <* 0.01 versus the control group; *p < 0.05, **p < 0.01 versus the model group; ▲*p <* 0.05 versus the CBZ group (*n* = 6 per group).

We used the MWM test to assess the effect of CDDP and its combination with CBZ on cognitive impairment at 90 d after surgery. In the place navigation test (Figure 3 and Table 1), all groups showed a decreasing trend in escape latency as the number of training days increased. The escape latencies were markedly longer in the model and CBZ groups on each training day than those in the control group (*p* < 0.05). The combined group also had longer escape latencies than the control group on the second (*p* < 0.05) day, whereas the CDDP and combined groups showed significantly quicker escape latencies during the training sessions than those recorded for the model group (*p* < 0.05). In the spatial probe trials (Figure 4(A,B)), the frequency of swimming across the platform in the model, CBZ, CDDP and combined groups was lower than in the control group (*p* < 0.05), whereas the frequency of crossing was significantly higher in the CDDP (*p* < 0.01) and combined groups (*p* < 0.05) than in the model group. The model and CBZ groups spent significantly less time in the target quadrant than the control group did (*p* < 0.05), whereas the CDDP and combined groups significantly improved their performance compared with the model group (*p* < 0.05). In addition, the CDDP group spent significantly more time in the target quadrant than the CBZ group did (*p* < 0.05). These results indicate that CDDP alone and in combination with CBZ could markedly improve learning and memory.

**Table 1.** Average escape latencies to find the hidden platform for each trial day in the MWM test.

Group	*n*	Latency at different times			
		Day 1	Day 2	Day 3	Day 4
Saline	6	67.4 ± 4.5	37.6 ± 4.2	26.9 ± 3.8	24.2 ± 2.7
Model	6	91.2 ± 6.3△	78.2 ± 5.0△△	59.5 ± 5.5△△	50.6 ± 4.9△△
CDDP	6	78.9 ± 6.4	52.1 ± 5.1**	39.5 ± 2.6**	33.0 ± 2.8*
CBZ	6	86.3 ± 4.3	75.1 ± 5.0△△	49.5 ± 4.5△△	45.7 ± 4.1△
CDDP + CBZ	6	81.7 ± 3.5	63.1 ± 4.1△	42.9 ± 1.8*	35.5 ± 4.4

Results are presented as mean ± SEM. △*p <* 0.05. △△*p <* 0.01 versus the control group. *p < 0.05. **p < 0.01 versus the model group.

### Effect of CDDP and its combination with CBZ on KA-induced neuron death

**Figure 5.**
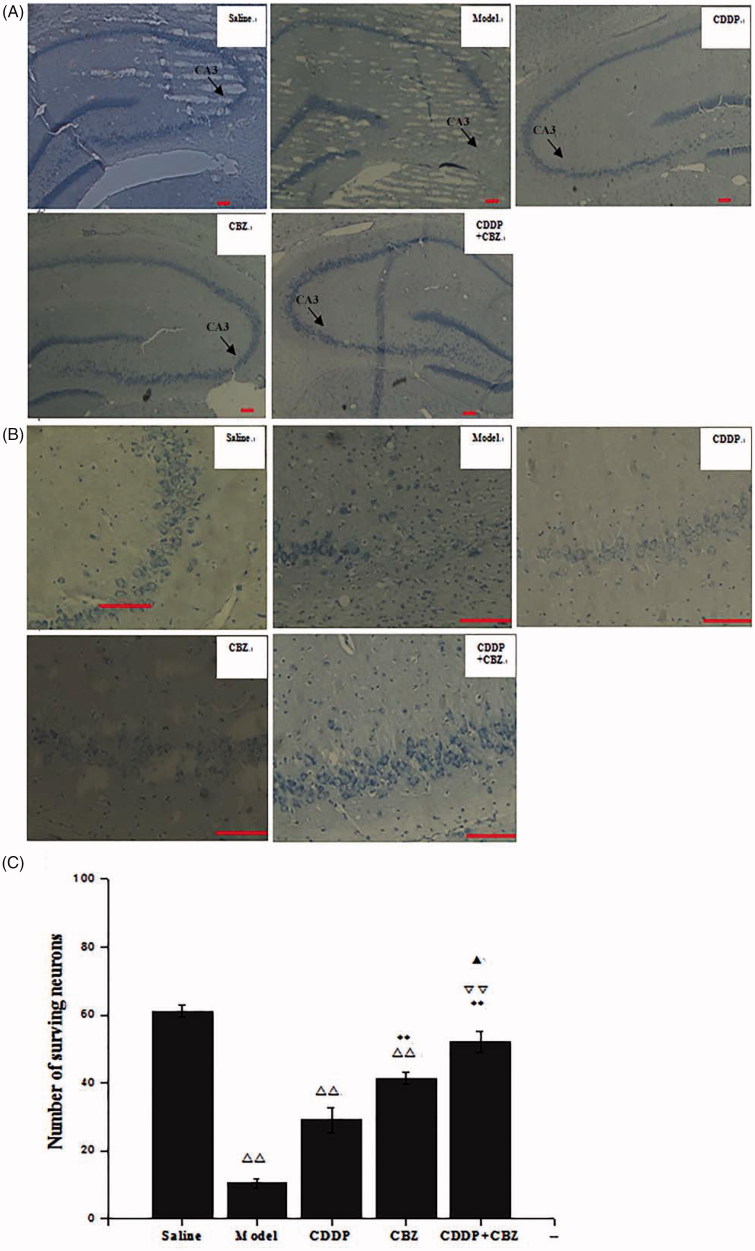
Effect of CDDP and its combination with CBZ on KA-induced neuron death (A) Nissl staining was used to assess the neuronal damage of hippocampus (magnification ×50). The arrowheads show the position of the hippocampal CA3 area. (B) The neuronal loss in the hippocampal CA3 region (magnification ×200). (C) The number of surviving neurons in the hippocampal CA3 region was counted. The results are presented as means ± SEM. △△*p <* 0.01 versus the control group; **p < 0.01 versus the model group; ▽▽*p <* 0.01 versus the CDDP group; ▲*p <* 0.05 versus the CBZ group (*n* = 6 per group). Scale bars: 100 μm.

Hippocampal neuronal damage was assessed by Nissl staining at 90 d after surgery. The number of neurons in the hippocampus CA3 area was counted. We found that KA induced neuronal cell loss in this area. The combined group showed no obvious neuronal loss when compared with the control group, whereas the other groups showed extensive loss (*p* < 0.01) (Figure 5(A–C)). The CBZ and combined groups had significantly less neuronal damage in the CA3 region than the model group (*p* < 0.01). Furthermore, the number of surviving neurons in the combined group was significantly greater than that in the CDDP (*p* < 0.01) and CBZ (*p* < 0.05) groups. The results show that CBZ and CDDP combined with CBZ clearly reversed the loss of hippocampal neurons in the CA3 area induced by KA, with the combined group showing the most improvement.

### Effect of CDDP alone and in combination with CBZ on the expression of GDNF and Bcl-2/Bax in the hippocampal CA3 region

**Figure 6.**
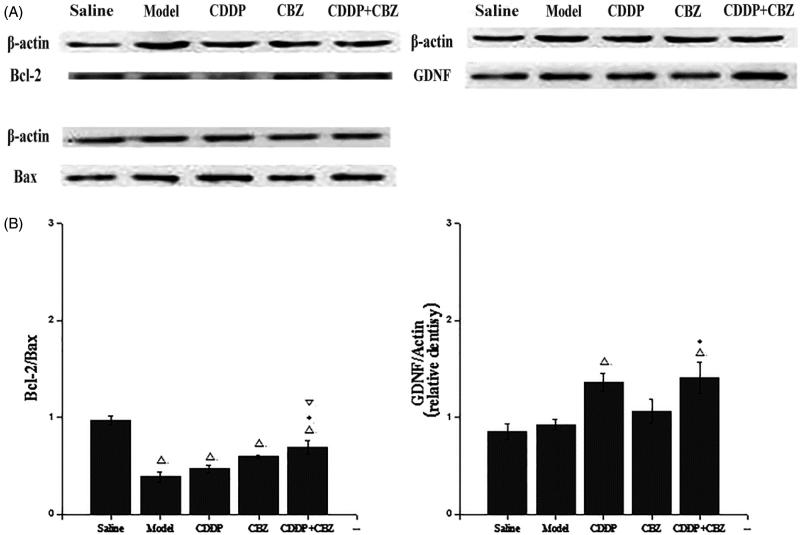
Effect of CDDP and its combination with CBZ on the expression of GDNF and Bcl-2/Bax in the hippocampal CA3 region. (A) Western blotting was used to evaluate protein expression of GDNF, Bcl-2 and Bax, and β-actin was used as an internal control. (B) Densitometry analysis was performed using Bio-Rad Quantity One software. The relative band density of GDNF and Bcl-2/Bax are shown in the bar diagram. The results are presented as means ± SEM. △*p < *0.05 versus the control group; *p < 0.05 versus the model group; ▽*p <* 0.05 versus the CDDP group (*n* = 6 per group).

GDNF and Bcl-2/Bax expression in the hippocampal CA3 area was evaluated by western blotting at 90 d after surgery. As shown in Figure 6(A,B), GDNF expression in the CDDP and combined groups showed a clear increase over the control group (*p* < 0.05), whereas the expression of GDNF was significantly greater in the combined groups than in the model group (*p* < 0.05). The expression of Blc-2/Bax in all groups was lower than in the control group (*p* < 0.05). Compared with the model group, the combined treatment significantly suppressed the KA-induced decrease of Blc-2/Bax expression (*p* < 0.05). These results indicate that CDDP in combination with CBZ can remarkably up-regulate the expression of GDNF protein and the level of Bcl-2/Bax in the hippocampal CA3 region of KA-induced rats.

## Discussion

Rats that are given intracerebral or systemic kainic acid (KA) develop neuropathological changes such as neurodegeneration, severe status epilepticus (SE), neuronal cell death and memory loss and experience chronic, spontaneous, recurrent seizures (Contractor et al. 2001; Zagulska-Szymczak et al. 2001). This model of KA-induced epilepsy closely resembles that found in humans (Sperk 1994) and has become the most widely accepted animal model for the study of human TLE (Hong et al. 1993; Loscher 1997). In this study, a rat model of TLE was established by intrahippocampal KA injection. We found that in the KA-induced TLE rat model, neither CDDP nor CBZ monotherapy could effectively control epileptogenesis or seizure severity, but CDDP exerted a significant cognition-protective effect with the extension of treatment time. However, combined administration of CDDP and CBZ produced a positive interaction effect. This combination of Chinese and Western drug therapy decreased the seizure severity and SRS frequency, markedly improved cognitive impairment and significantly decreased neuronal damage by up-regulating the expression of GDNF proteins and the level of Bcl-2/Bax in the hippocampal CA3 region. This combination therapy showed a better protective effect than monotherapy with either CDDP or CBZ.

TLE is often associated with cognitive dysfunction (Tavakoli et al. 2011; El-Kattan et al. 2013). The causes of this comorbidity mainly relate to damage to the temporal lobe, particularly the hippocampus and amygdala (Bell and Giovagnoli 2007; Kennepohl et al. 2007). Other reasons are the long-term administration of AEDs and seizure-related factors such as the duration of TLE (Sayin et al. 2004). In our MWM experiments, rats in both the model and CBZ groups had remarkably longer escape latencies and lower scores for the frequency of platform crossing and time in the target quadrant than those in the control group. Our results are consistent with studies that have found significant cognitive impairment associated with TLE (Tavakoli et al. 2011; El-Kattan et al. 2013). In addition, we found that monotherapy with CBZ did not improve cognitive impairment in KA-treated rats. However, treatment with CDDP alone or in combination with CBZ not only markedly decreased the mean escape latencies but also increased the platform-crossing frequency and time in the target quadrant, suggesting that CDDP can improve cognitive impairment in a rat model of KA-induced TLE.

A pathological characteristic of TLE is progressive damage to and pronounced neuronal loss in the hippocampus (Kapur 2003; Yamamoto et al. 2006). Cognitive dysfunction is also related to hippocampal neuronal cell loss (Detour et al. 2005; Hermann et al. 2006). In the Bcl-2 gene family, Bax is a pro-apoptotic protein and Bcl-2 is an anti-apoptotic protein. Their role is to maintain homeostasis between cellular survival and demise (Chen et al. 2001). The ratio of Bcl-2 to Bax determines apoptosis induction (Woo et al. 2000; Salakou et al. 2007). In this study, the CA3 regions of the hippocampus showed no obvious neuronal loss in the combined group, whereas more neurons were lost in the other group compared with the control group. Moreover, the Bcl-2/Bax ratio in the KA-treated groups was lower than that in the control group, whereas combined treatment significantly suppressed this decrease compared with the model group. These results indicate that KA-induced neuronal cell apoptosis in the hippocampus CA3 region is probably the main reason for this neuronal cell death. Treatment with a combination of CDDP and CBZ can reverse the damage to hippocampal neurons by protecting neuronal cells from apoptosis.

GDNF is widespread in the rat and human central nervous system and is also present in the hippocampus (Schmidt-Kastner et al. 1994), which is crucial for the survival and regeneration of neurons after epilepsy seizure (Airaksinen and Saarma 2002). It has been shown that treatment with rhGDNF can inhibit KA-induced seizures and also protect neuronal cell loss in the hippocampal, amygdaloidal and thalamic regions (Martin et al. 1995). To assess the neuroprotective effect of CDDP and CBZ in a KA-induced TLE model, we further investigated the expression of the neurotrophic factor GDNF in the CA3 region of the hippocampus. We found that CDDP combined with CBZ led to an obvious increase in GDNF expression compared with the control and model groups, indicating that this combination can up-regulate the expression of GDNF. Given these findings, we suggest that inhibited apoptosis due to CDDP combined with CBZ may be partly correlated with the up-regulation of GDNF expression in the CA3 area of the hippocampus.

In conclusion, our findings support the use of CDDP as an adjuvant drug for the treatment of TLE and cognitive deficit. Combined administration of CDDP with CBZ was more effective for control of epileptogenesis, protection against cognitive impairment and inhibition of hippocampal neuronal loss in a model of KA-induced TLE. Its mechanism might be related to anti-apoptosis and up-regulation of GDNF expression level. This combination, if applicable to individual clinical therapy, may provide a potential novel therapeutic option to enhance the treatment of patients with TLE.

## Supplementary Material

Jiao_Haisheng_et_al_supplemental_content.zip
