# Supplementary material for: Protective effect of compound Danshen (Salvia miltiorrhiza) dripping pills alone and in combination with carbamazepine on kainic acid-induced temporal lobe epilepsy and cognitive impairment in rats
Source: Pharm Biol. 2018 Mar 21;56(1):217–24. doi: 10.1080/13880209.2018.1432665 (PMC6130614; doi:10.1080/13880209.2018.1432665)
Supplement: Jiao_Haisheng_et_al_supplemental_content.zip [file IPHB_A_1432665_SM9527.zip › Final suppl file.docx]

HPLC Fingerprints of compound Danshen dripping pills

**Objective**

In this supplementary information, we used a validated HPLC method in combination with the similarity analysis (Sun et al., 2013) to establish an HPLC fingerprint of compound Danshen dripping pills (CDDP), determine the contents of main active compounds in CDDP and assess the quality of CDDP used in the study “Protective effect of compound Danshen (Salvia miltiorrhiza) dripping pills alone and in combination with carbamazepine on kainic acid-induced temporal lobe epilepsy and cognitive impairment in rats”.

**Method**

**Sample solutions**

Ten pills of CDDP (27 mg/pill; Tianjin Tasly Co., Ltd., China, #151204) were accurately weighed and extracted with 50 mL ethanol:water (1:1, v/v) for 1 h. Then the filtrate was concentrated to approximately 10 mL under the reduced pressure in a rotary evaporator. Finally, ethanol:water (1:1, v/v) was added to make the volume of 25 mL.

**Standard solutions**

Protocatechualdehyde (PHA) of 5.0 mg was accurately weighed and 50% (V/V) methanol was added to make 25 mL exactly to serve as the standard solution after shaking. Danshensu (DSS) and salvianolic acid B (SAB) solutions were prepared in the same way. PHA, DSS and SAB were all purchased from the National Institute for the Pharmaceutical and Biological Products (Beijing, China, for quantified grade).

**Chromatographic condition**

An Agilent 1100 series system (Agilent Corporation, USA) with a diode-array detector was used for chromatographic analysis on an Agilent Zorbax Eclipse XDB-C18 column (250 mm × 4.6 mm, 5 μm, Agilent Corporation). The mobile phase was composed of solutions A (1% acetic acid in water) and B (methanol with 1% acetic acid) by using a gradient program of 10-32% B in 0-15 min, 32-45% B in 15-30 min, and 45-80% B in 30-50 min. There was a 15 min re-equilibration between individual runs to recover the initial conditions. The column temperature was maintained at 30°C and the detection wavelength was set at 290 nm. The flow rate was 1.0 mL/min and the injection volume was 10 μL.

**Results**

[Fig. 1](https://www.dovepress.com/protective-effect-of-huang-gan-formula-in-56-nephrectomized-rats-by-de-peer-reviewed-fulltext-article-DDDT#F1)A shows the HPLC fingerprint chromatogram of ten different batches of CDDP. The software “Similarity Evaluation System for Chromatographic Fingerprint of Traditional Chinese Medicine (Version 2012)” was used to evaluate the similarities between 10 batches of CDDP (including test batch, lot No.: 151204). 20 co-possessing peaks were collected and used for the similarity analysis. The reference fingerprint of CDDP was set up in terms of calculating the means of the 10 batch of CDDP fingerprint signals, and it severed as the norm to evaluate their quality results. The similarity degree of the test batch was 0.992 and the similarity degrees of 10 batches were all over 0.98. A total of three major compounds were identified and quantified by comparing reference substance. Their names and contents (mg/g) were DSS (12.05 ± 0.26), PHA (3.74 ± 0.18) and SAB(5.65 ± 0.31), as shown in Fig. 1B.

**Conclusion**

This study has determined the contents of three main active components in CDDP and proved the quality of the test batch by using the HPLC fingerprint.

**References**

Sun G, Song Y, Li L, et al. 2013. Quickly quantifying the dissolution fingerprints of compound Danshen dropping pill by HPLC. Ann Transl Med. 1:16.


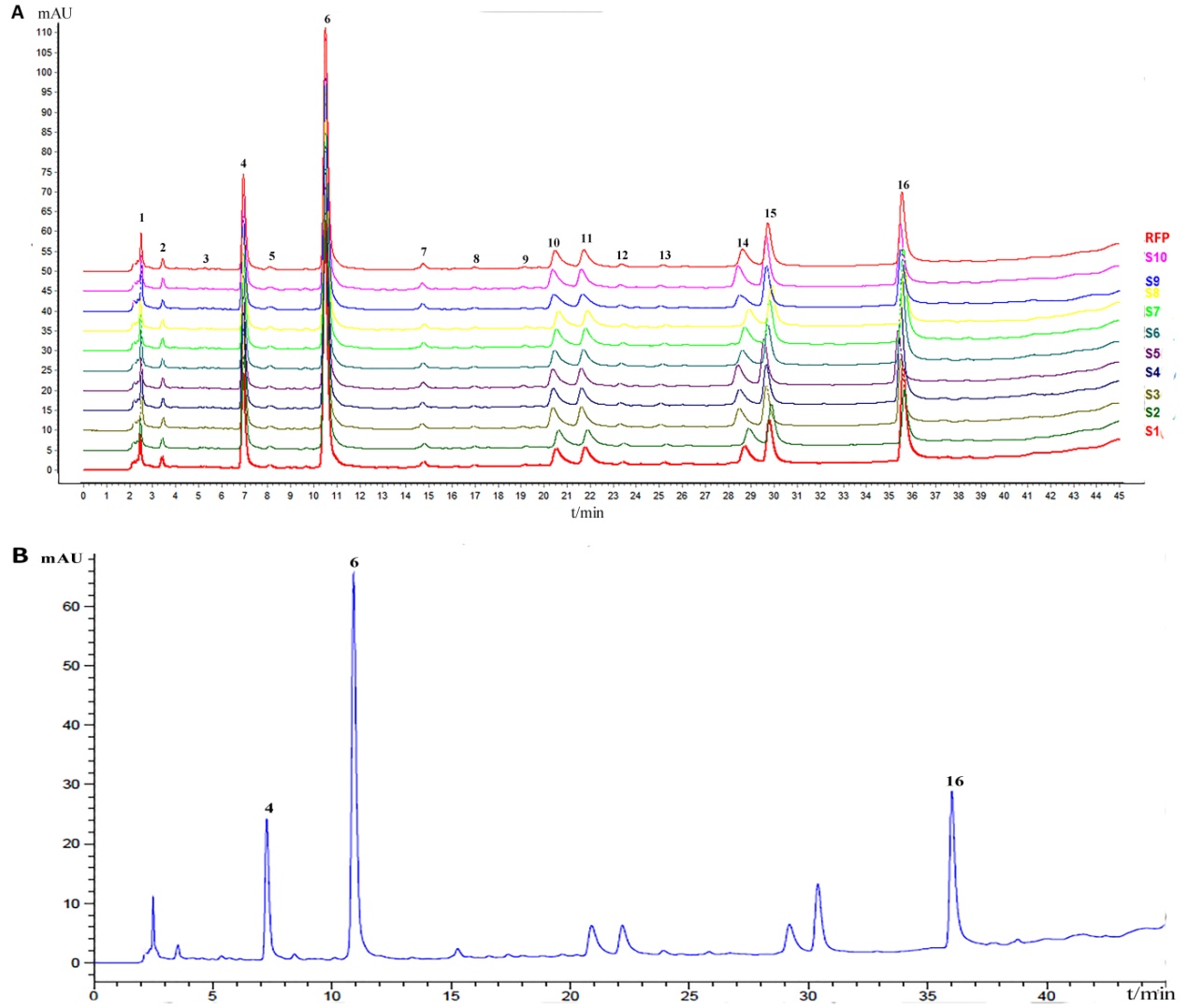


Fig. 1 shows the HPLC fingerprints of CDDP. (A) The HPLC fingerprints of 10 batches of CDDP and their average reference fingerprint (RFP). The test batch was labeled as S1. Twenty marked peaks were co-possessing in CDDP fingerprint. (B) The identification of DSS (4), PHA(6) and SAB (16) was shown in the chromatogram.
